# The secondary bile acid isoursodeoxycholate correlates with post-prandial lipemia, inflammation, and appetite and changes post-bariatric surgery

**DOI:** 10.1016/j.xcrm.2023.100993

**Published:** 2023-04-05

**Authors:** Panayiotis Louca, Abraham S. Meijnikman, Ana Nogal, Francesco Asnicar, Ilias Attaye, Amrita Vijay, Afroditi Kouraki, Alessia Visconti, Kari Wong, Sarah E. Berry, Emily R. Leeming, Olatz Mompeo, Francesca Tettamanzi, Andrei-Florin Baleanu, Mario Falchi, George Hadjigeorgiou, Jonathan Wolf, Yair I.Z. Acherman, Arnold W. Van de Laar, Victor E.A. Gerdes, Gregory A. Michelotti, Paul W. Franks, Nicola Segata, Massimo Mangino, Tim D. Spector, William J. Bulsiewicz, Max Nieuwdorp, Ana M. Valdes, Cristina Menni

**Affiliations:** 1 Department of Twin Research & Genetic Epidemiology, King’s College London, SE1 7EH London, UK; 2 Department of (Experimental) Vascular Medicine, Amsterdam University Medical Centre (UMC), Amsterdam, the Netherlands; 3 Department CIBIO, University of Trento, Trento, Italy; 4 Nottingham NIHR Biomedical Research Centre at the School of Medicine, University of Nottingham, NG5 1PB Nottingham, UK; 5 Inflammation, Recovery and Injury Sciences, School of Medicine, University of Nottingham, NG5 1PB Nottingham, UK; 6 Metabolon, Research Triangle Park, Morrisville, NC, USA; 7 Department of Nutritional Sciences, King’s College London, London, UK; 8 Zoe Limited, London, UK; 9 Department of Surgery, Spaarne Gasthuis, Hoofddorp, the Netherlands; 10 Lund University Diabetes Center, Lund University, Malmö, Sweden; 11 Department of Clinical Sciences, Lund University, Malmö, Sweden; 12 NIHR Biomedical Research Centre at Guy’s and St Thomas’ Foundation Trust, SE1 9RT London, UK

**Keywords:** bile acids, post-prandial, triglycerides, bariatric surgery, liver function

## Abstract

Primary and secondary bile acids (BAs) influence metabolism and inflammation, and the gut microbiome modulates levels of BAs. We systematically explore the host genetic, gut microbial, and habitual dietary contribution to a panel of 19 serum and 15 stool BAs in two population-based cohorts (TwinsUK, n = 2,382; ZOE PREDICT-1, n = 327) and assess changes post-bariatric surgery and after nutritional interventions. We report that BAs have a moderately heritable genetic component, and the gut microbiome accurately predicts their levels in serum and stool. The secondary BA isoursodeoxycholate (isoUDCA) can be explained mostly by gut microbes (area under the receiver operating characteristic curve [AUC] = ∼80%) and associates with post-prandial lipemia and inflammation (GlycA). Furthermore, circulating isoUDCA decreases significantly 1 year after bariatric surgery (β = −0.72, p = 1 × 10^−5^) and in response to fiber supplementation (β = −0.37, p < 0.03) but not omega-3 supplementation. In healthy individuals, isoUDCA fasting levels correlate with pre-meal appetite (p < 1 × 10^−4^). Our findings indicate an important role for isoUDCA in lipid metabolism, appetite, and, potentially, cardiometabolic risk.

## Highlights

• Levels of the secondary bile acid isoUDCA are determined by the gut microbiome
• isoUDCA levels associate with higher post-prandial lipemia and fasting appetite
• Higher isoUDCA levels correlate with worse liver function enzyme values
• isoUDCA levels decrease post-bariatric surgery and after fiber supplementation

## Graphical abstract

**Figure:**
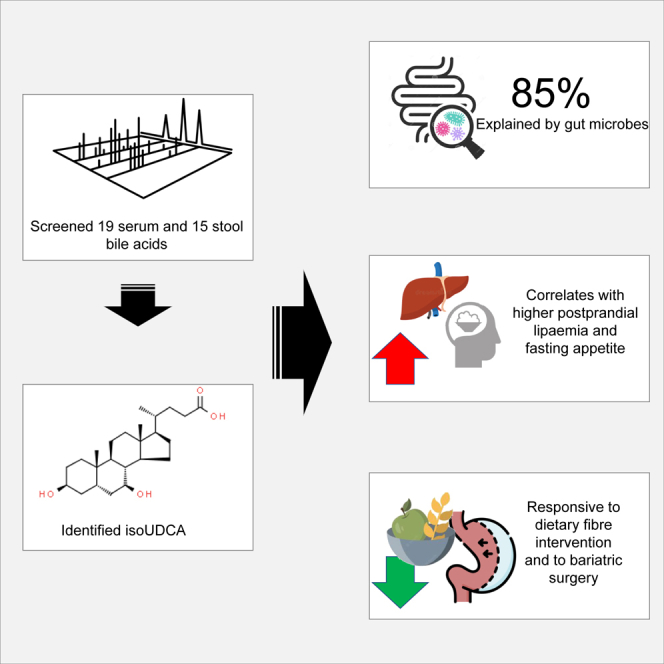

## Teaser

Weight-independent effects of bariatric surgery on metabolism are attributed to changes in bile acids and the gut microbiome. The results of Louca et al. suggest that isoUDCA levels are linked to hypertriglyceridaemia and increased appetite. Reduction in its levels may mediate increased satiety and improved lipemic control post-bariatric surgery.

## Introduction

Bile acids (BAs) are increasingly seen as key mediators of the effect of the gut microbiome on cardiometabolic and gut health.^1^^,^^2^ BAs influence energy expenditure, glycemia, lipemia, insulin sensitivity, inflammation, the immune system,^3^^,^^4^^,^^5^ and the role of chronic insomnia on cardiometabolic disorders.^6^^,^^7^^,^^8^^,^^9^ They can also modify gut microbial composition through their antimicrobial activities.^10^

Fasting BA concentrations are typically low, but levels increase during metabolic stress and during a post-prandial state, activating receptors.^11^ Lipid post-prandial responses have been linked with liver fat content^12^^,^^13^ and an increased risk of cardiovascular disease (CVD) and mortality.^14^^,^^15^ The link between a highly individualized post-prandial lipemic response and the gut microbiome may be explained by secondary BAs, which are formed by the gut microbiome and then influence lipid metabolism.

Bariatric surgery is the most effective and durable treatment for individuals with class III obesity (BMI > 35 kg/m^2^), which elicits changes in metabolic health,^16^^,^^17^ appetite,^18^^,^^19^ and several comorbidities. Bariatric surgery is also understood to increase primary BA concentrations, with evidence from rodent models^17^ and human studies,^20^ and this is most prominent during a post-prandial state.^11^ The improvement in metabolic function post-bariatric surgery may be partly explained by changes in BA concentrations.

Thus, to improve our understanding of these relationships, the aim of this study was to carry out a comprehensive screening of the host genetic, gut microbiome, and habitual dietary contributions to fecal and serum levels of BAs in two UK-based population cohorts. We further investigated, in a posteriori analysis, the association between BAs and post-prandial lipemia, liver function, and inflammation, and we assessed changes in BA concentrations post-bariatric surgery and post-dietary interventions.

## Results

**Figure 1:**
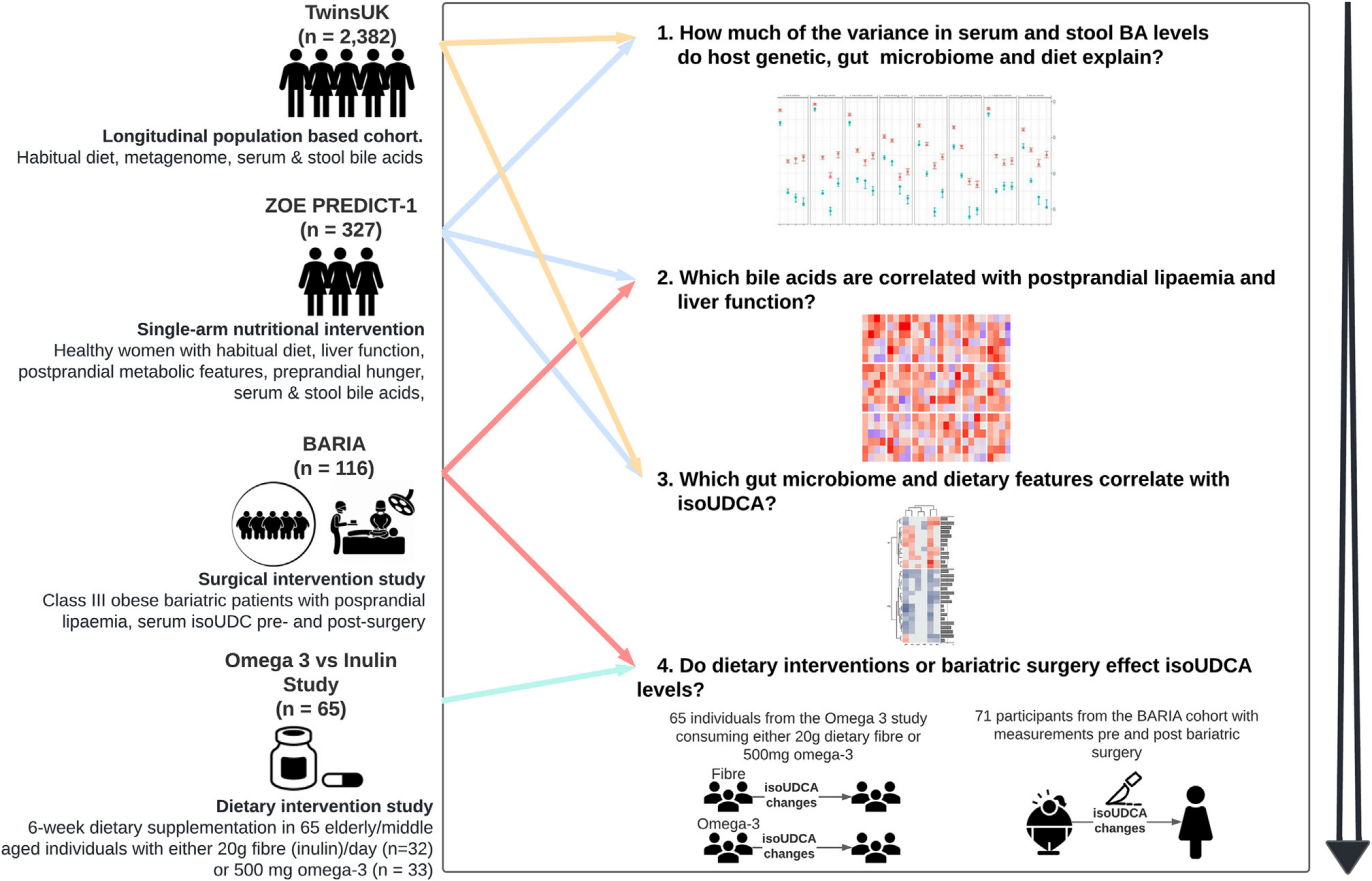
Schematic representation of the study In this schematic representation of the study, we highlight for each step the research question we want to answer, the analysis workflow, and the data used. We first aimed to estimate how much of the variance in serum and fecal bile acids is explained by the host genome, the gut microbiome, and diet in the TwinsUK and ZOE PREDICT-1 cohorts (step 1). We then investigated which primary and secondary bile acids are correlated with post-prandial lipemia, inflammation, and liver function in ZOE PREDICT-1 and replicated the top hit in the BARIA study (step 2). Finally, we further identified the gut microbiome and dietary features correlating with the BA (isoUDCA) associated with post-prandial lipemia, inflammation, and liver function in TwinsUK and ZOE PREDICT-1 (step 3) and determined how much dietary intervention (Omega-3 and Fiber Intervention to Improve Metabolic Health study) and bariatric surgery (BARIA study) affect its circulating levels.

A flowchart of the study design is presented in Figure 1.

The descriptive characteristics of the study populations are presented in Table S1. We included 2,382 individuals from TwinsUK and 327 female individuals from the ZOE PREDICT-1 study, with primary and secondary BAs measured in serum (n = 19) and stool (n = 15) (Table S2) by Metabolon. The average age in TwinsUK was 58.9 (14.6) and in ZOE PREDICT-1 was 53.8 (7) years, while the average BMIs were 26 (5) and 26.3 (5.6) kg/m^2^, respectively.

The correlations between BAs in TwinsUK and ZOE PREDICT-1 are presented in Figure S1. The post-prandial changes (at 30 min, 2 h, and 4 h) in circulating BAs in ZOE PREDICT-1 are depicted in Figure S2. We found that circulating levels of primary and secondary BAs, on average, increase after a meal challenge.

### Host genetic, gut microbiome, and dietary contributions

**Figure 2:**
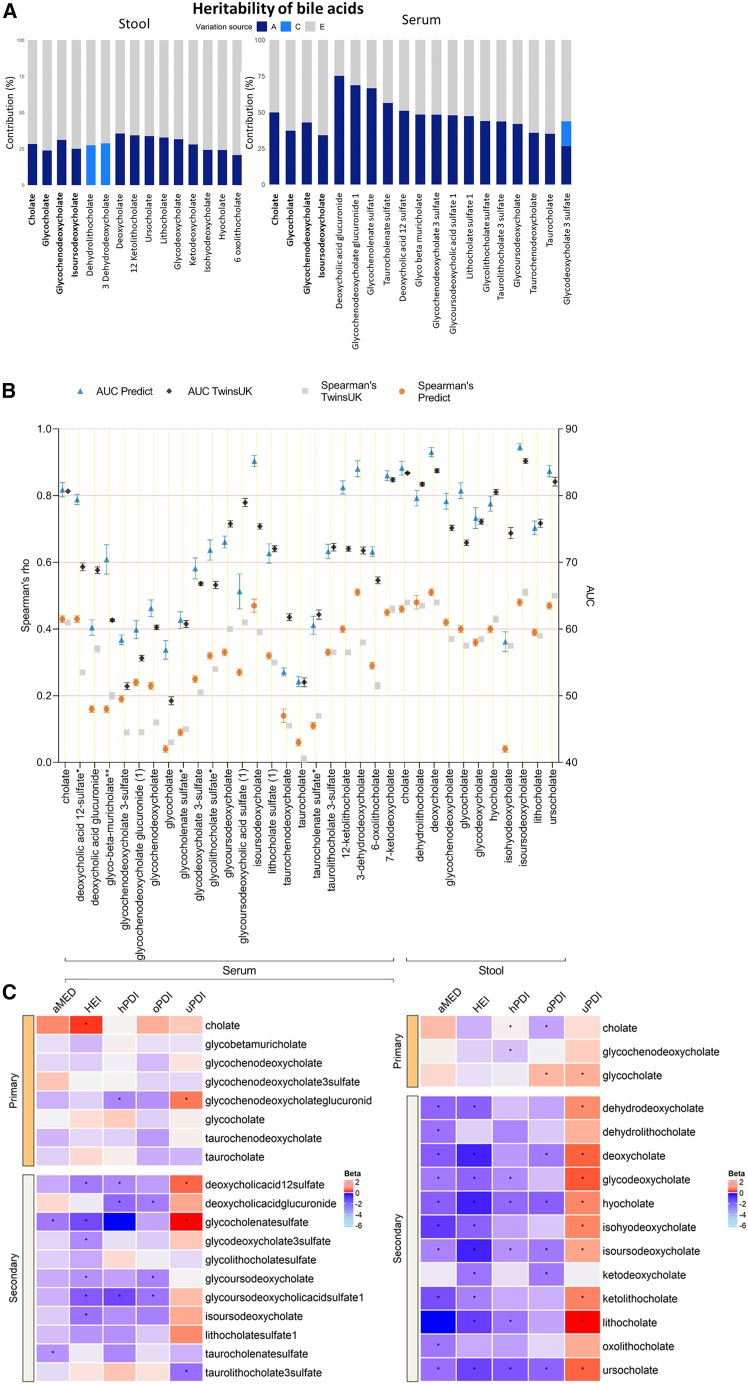
Host genetics, gut microbiome, and dietary contributions to serum and stool BA concentrations in the TwinsUK and ZOE PREDICT-1 studies (A) Heritability estimates of BAs calculated in TwinsUK and ZOE PREDICT-1 participants. The A, C, and E labels indicate the percentage of variance attributed to the additive genetic factors, common/shared environmental factors, and unique environmental factors, respectively. Labels in bold show the BAs detected in both samples. (B) Prediction of the gut microbiota in BAs levels estimated by random forest regressors (using Spearman’s correlations) and classifiers (using AUC values) in 845 TwinsUK and 327 ZOE PREDICT-1 participants. Dark and light blue bars represent the mean AUC and the 95% confidence intervals across 5-fold for TwinsUK and ZOE PREDICT-1, respectively. Gray and orange bars indicate the mean and the 95% confidence intervals of the Spearman’s correlations between the real value of each BA and the value predicted by the models across 5-fold in TwinsUK and ZOE PREDICT-1, respectively. (C) Random effects inverse variance meta-analyzed coefficients from linear models in TwinsUK and ZOE PREDICT-1 between serum and stool BAs and dietary quality measures (oPDI, original plant diversity index; uPDI, unhealthy plant diversity index; hPDI, healthy plant diversity index; aMED, alternative Mediterranean diet score; HEI, Healthy Eating Index). Significant correlation is denoted by ∗p < 0.05.

To test whether host genetics influences primary and secondary BAs, we estimated heritability, taking advantage of the twin structure in our data by using structural equation modeling and pulling together TwinsUK and ZOE PREDICT-1 (n = 2,539, 654 monozygotic [MZ] pairs, 380 dizygotic [DZ] pairs, and 471 singletons). We decomposed the observed phenotypic variance into three latent sources of variation: additive genetic variance (A), shared/common environmental variance (C), and non-shared/unique environmental variance (E).^21^ The AE model was the best fitting model for 31 of the 34 BAs. For genetic factors, heritability estimates ranged from 21% (95% confidence interval [CI]: 13.8%, 27.6%) for 6-oxolithocholate to 75% (95% CI: 72.1%, 78.1%) for deoxycholic acid glucuronide, as depicted in Figure 2A. Given the major role of the gut microbiome in post-translational modification of BAs, we investigated the gut microbiome contribution to primary and secondary serum and stool BA levels using random forest models, which were trained on gut microbiota abundances to predict each circulating and fecal BA. The model performance was evaluated using the area under the receiver operating characteristic curve (AUC) for the classifiers and Spearman’s correlations between the predicted and real values for the regressors. In TwinsUK, all the BAs in stool, except 6-oxolithocholate, presented a mean Spearman’s correlation >0.36 and AUC >71% over the cross-validation folds (Figure 2B), with isoursodeoxycholate (isoUDCA) presenting the strongest association (AUC [95% CI]: 85.2% [84.8, 85.6]; ρ [95% CI]: 0.48 [0.47, 0.49]). On the other hand, the prediction performance in serum BAs based on AUC values ranged from 49.2% (95% CI: 48.6, 49.8) for glycocholate to 80.7% (95% CI: 80.4, 80.8) for cholate (Figure 2B). In serum, isoUDCA showed the fourth strongest association (AUC [95% CI]: 75.4% [75, 75.8]; ρ [95%CI]: 0.39 [0.38, 0.41]). Results were validated in the ZOE PREDICT-1 cohort (Table S3).

We further investigated the correlation between serum and stool BAs and the quality of habitual diet as measured by the healthy eating index (HEI)^22^ by the alternative Mediterranean diet score (aMED)^23^ and by the plant diversity indices (PDIs) (including the original PDI [oPDI], the healthy PDI [hPDI], and the unhealthy PDI [uPDI]).^24^ As depicted in Figure 2C, the effects observed were modest, but we found a consistent trend with individuals adhering to a healthier diet (based on scores of the oPDI, hPDI, aMED, and HEI) having lower levels of both stool and serum BAs concentrations.

### BAs and post-prandial lipemia

**Figure 3:**
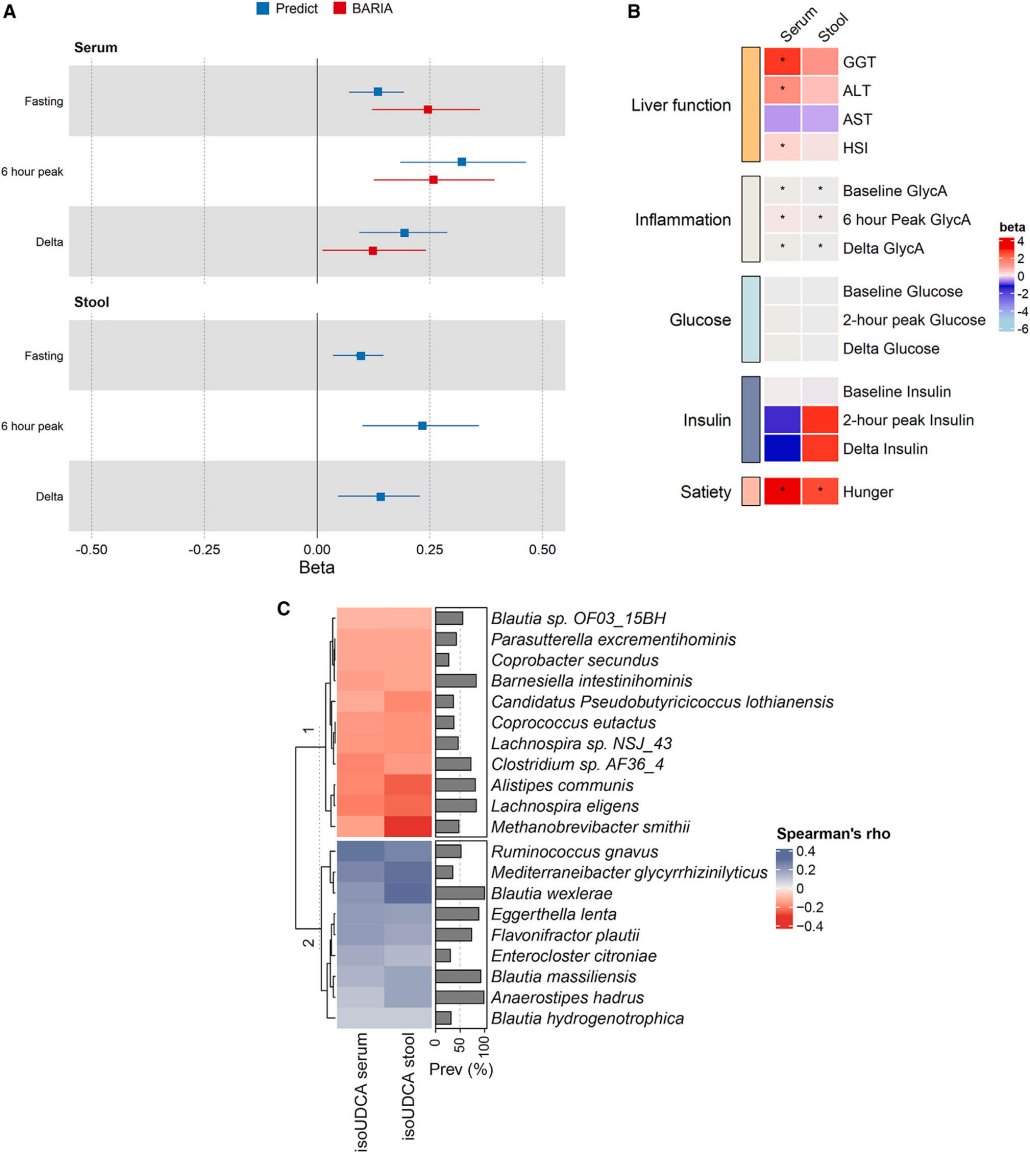
Relationship between isoursodeoxycholate, metabolic health, and the gut microbiome Association between fasting serum and stool isoUDCA levels and (A) fasting triglycerides, triglyceride peak, and post-prandial triglyceride change in the ZOE PREDICT-1 cohort and in pre-surgery BARIA study participants (serum isoUDCA only) (for δ triglycerides in BARIA, we investigated the correlation between isoUDCA and δ first and third tertiles); (B) liver function parameters, basal and post-prandial metabolic factors (inflammation, glucose, insulin), and appetite in ZOE PREDICT-1; and (C) single gut microbial species in TwinsUK and ZOE PREDICT-1. Partial Spearman’s correlations calculated adjusting for age, BMI, and sex assigned at birth in 845 TwinsUK participants. Only the characterized species with a prevalence >20% that had significant correlations (FDR < 0.05) and presented the same directional effects in TwinsUK and ZOE PREDICT-1 are shown. Species are hierarchically clustered (complete linkage, Euclidean distance).

We further investigated the association between fasting serum and stool BAs and post-prandial lipemia in the 327 females that undertook the ZOE PREDICT-1 standardized test meal challenge.^13^ After adjusting for age, BMI, and multiple testing (q < 0.05), fecal abundances of the secondary BAs isoUDCA (β [95% CI], 0.23 [0.1, 0.36], p = 5.52 × 10^−4^), ursocholate (β [95% CI], 0.26 [0.12, 0.39], p = 1.86 × 10^−4^), and deoxycholate (β [95% CI], 0.21 [0.08, 0.33], p = 1.1 × 10^−3^) were associated with higher post-prandial lipemia (Figure S3; Table S4), measured as the highest triglyceride concentration in the 6 h following the test meal challenge. In serum, we found only fasting levels of isoUDCA to correlate with post-prandial lipemia (β [95% CI], 0.32 [0.18, 0.46], p = 7.36 × 10^−6^), after adjusting for age, BMI, and multiple testing (false discovery rate [FDR] < 0.05) (Figure 3A). Results remained consistent when looking at the δ change from fasting triglycerides and when further accounting for dietary fat intake as a percentage of total energy intake. Since only isoUDCA both in serum and stool was consistently associated in serum and stool with post-prandial lipemia, we focused on this BA. We first replicated the serum lipemia associations for isoUDCA in 116 individuals with class III obesity from the BARIA cohort: post-prandial = β (95%CI) = 0.26 (0.13, 0.39), p = 4.1 × 10⁻^4^, and fasting = β (95% CI) = 0.24 (0.12, 0.36), p = 1.81 × 10^−4^ (Figure 3A), adjusting for age, sex assigned at birth, and BMI. We also find a significant difference in change from fasting triglycerides when looking at tertiles of change (β [95% CI] = 0.13 [0.01, 0.24], p = 6.84 × 10^−3^) (Figure 3A). The association between circulating isoUDCA and fasting triglyceride was also replicated in the TwinsUK cohort (β [95% CI] = 0.07 [0.04, 1.00], p < 1 × 10⁻^4^).

In the ZOE PREDICT-1 cohort, both fasting serum and stool levels of isoUDCA were also associated with post-prandial inflammation, as determined by peak GlycA over 6 h (stool = β [95% CI] = 0.04 [0.01, 0.06], p = 7.24 × 10^−3^; fasting serum = β [95% CI] = 0.07 [0.04, 0.11], p = 1.64 × 10^−5^) but not with post-prandial changes in insulin or glucose (Figure 3B). Moreover, we report positive associations between serum isoUDCA and the hepatic steatosis index (HSI), a calculated measure predictive of hepatic steatosis^25^ (β [95% CI] = 0.34 [0.11, 0.57], p = 3.54 × 10^−3^), and the liver function enzymes alanine aminotransferase (ALT) (β [95% CI] = 1.36 [0.2, 2.51], p = 2.16 × 10^−2^) and γ-glutamyl transferase (GGT) (β [95% CI] = 2.74 [0.77, 4.72], p = 6.84 × 10^−3^). However, no significant association was detected between serum isoUDCA and aspartate transaminase (AST) levels (Figure 3B) nor between fecal isoUDCA and liver function tests or HSIs.

### Host genetic, microbiome, and dietary contributions to isoUDCA levels

As shown in Figure 2, isoUDCA is mainly environmentally determined, with the gut microbiome able to accurately predict its levels in serum and stool. We therefore further ran Speaman’s correlations adjusted for age, BMI, sex assigned at birth, and multiple testing to identify the most contributing species to both serum and stool isoUDCA levels. In TwinsUK, we identified 324 and 283 gut microbial species with a prevalence >20% to be significantly correlated (FDR < 0.05) with stool and serum isoUDCA, respectively, with 238 overlapping. Out of these, we identified *Blautia wexlerae* (stool: ρ = 0.3, p = 4.12 × 10^−17^; serum: ρ = 0.21, p = 9.62 × 10^−9^), *Eggerthella lenta* (stool: ρ = 0.19, p = 2.48 × 10^−7^; serum: ρ = 0.2, p *=* 9.46 × 10^−8^), and *Flavonifractor plautii* (stool: ρ = 0.17, p *=* 4.35 × 10^−6^; serum: ρ = 0.2, p *=* 8.5 × 10^−8^) to be positively correlated, whereas negative correlations included species such as *M. smithii* (stool: ρ = −0.28, p = 8.27 × 10^−17^; serum: ρ = −0.14, p = 6.74 × 10^−5^), and *C. eutactus* (stool: ρ = −0.16, p = 2.38 × 10^−6^; serum: ρ = −0.15, p = 8.15 × 10^−6^) (Figure 3C). Consistent results were obtained for ZOE PREDICT-1 (Figure S4). Figure 3C illustrates the characterized species with a prevalence >20% that had significant correlations (FDR < 0.05) and presented the same directional effects in both TwinsUK and ZOE PREDICT-1.

### Interventions to modify isoUDCA levels

As isoUDCA levels are environmentally determined, with the gut microbiome explaining 75.4% of the variance in its circulating levels and 85.2% of its fecal abundance with very little genetic contribution and its links to dietary intake, and having established a clear link between isoUDCA and post-prandial lipemia and liver function, we investigated the potential interventions in humans that could change isoUDCA levels.

**Figure 4:**
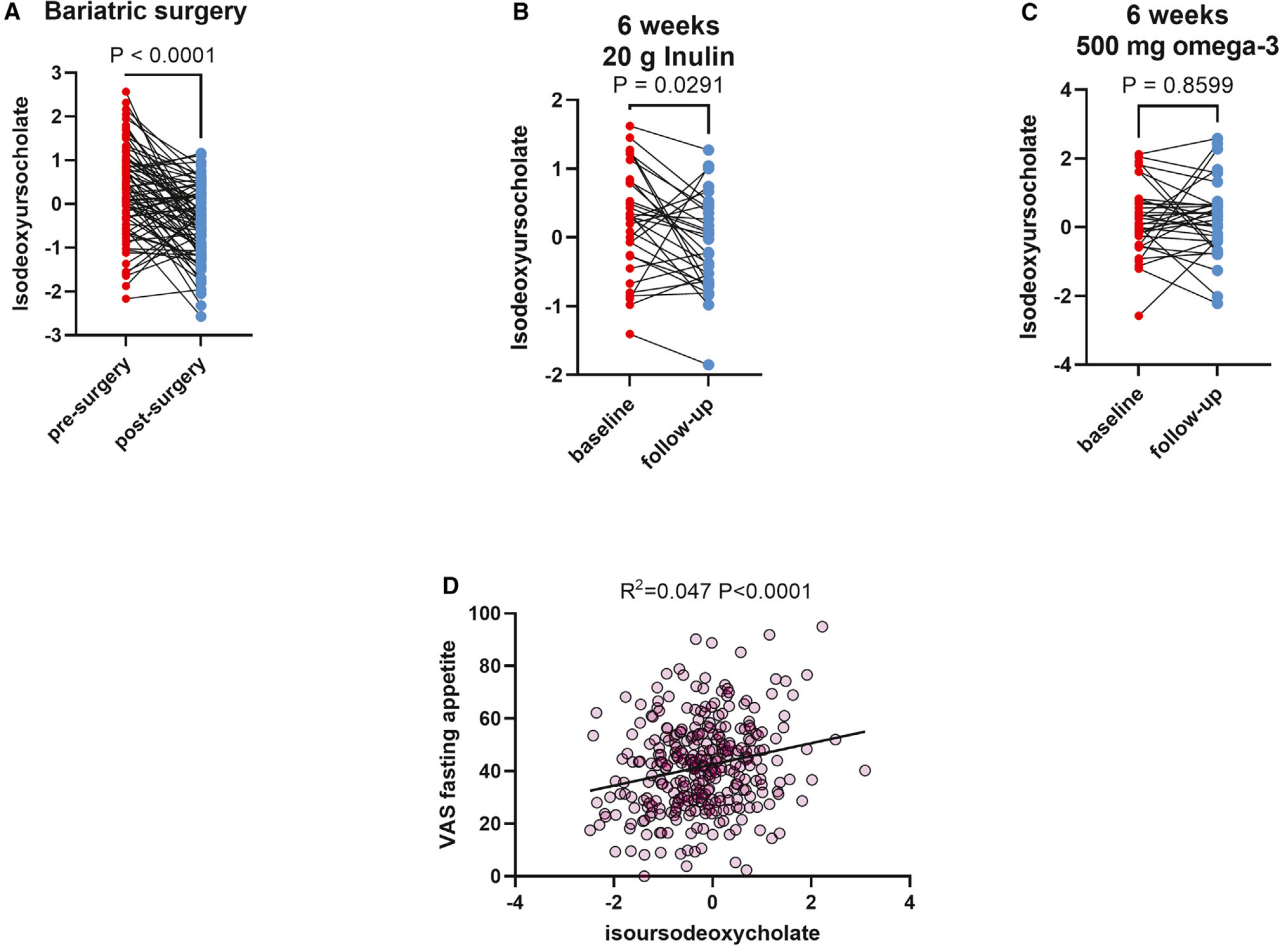
Interventions that modify isoUDCA (A) Effect of bariatric surgery on fasting levels of isoUDCA in the BARIA cohort (94% laparoscopic Roux-en-Y gastric bypass). isoUDCA levels were measured pre-surgery and 12 months after surgery. (B) Effect on fasting isoUDCA levels following a 6-week dietary supplementation intervention with 20 g/day inulin (C) or with 500 mg omega-3. (D) Correlation between fasting isoUDCA levels and self-reported fasting appetite in the ZOE PREDICT-1 study.

We tested this in an independent subset of TwinsUK (n = 65) who had participated in a 6-week dietary supplementation intervention and given either 20 g inulin or 500 mg omega-3^26^ and in 71 individuals who underwent bariatric surgery.^27^ We found that dietary supplementation of 20 g fiber (inulin) modestly reduced isoUDCA levels (mean of differences (follow up − baseline) = −0.37 [95% CI: −0.63, −0.04]; p < 0.029); however, 500 mg omega-3 brought about no changes over the course of a 6-week intervention (mean of differences (follow up − baseline) = +0.04 [95% CI: −0.41, +0.49]; p > 0.05) (Figure 4).

Randomized control trials have shown that various types of bariatric surgery^28^^,^^29^ can modify total basal and post-prandial BA levels. Accordingly, we explored whether isoUDCA levels changed pre- and post-bariatric surgery. In the BARIA cohort, of the samples included in this analysis, 71 underwent bariatric surgery (94% laparoscopic Roux-en-Y gastric bypass [LRYGB]) (average % of body weight change = −34.8%). We report significant changes in fasting circulating levels of isoUDCA 1 year post-surgery (Figure 4) (mean of differences (post-surgery − pre-surgery) = −0.72 [95% CI: −0.97, −0.47], p = 1 × 10^−5^) both overall and when stratifying by sex assigned at birth: in males, mean of differences (post-surgery − pre-surgery) = β (95% CI): −0.87 (−1.41, −0.43), p = 7 × 10^−4^, and in females, mean of differences (post-surgery − pre-surgery) = β (95% CI): −0.68 (−0.98, −0.39), p = 1 × 10^−5^.

### Changes in isoUDCA and changes in metabolic parameters and appetite

As isoUDCA is associated with poor metabolic health and changes post-intervention, we investigated if changes in isoUDCA following bariatric surgery or within the fiber arm of the omega-3/fiber intervention also correlated with changes in fasting triglyceride levels, body weight, and glucose. We found that changes in isoUDCA following bariatric surgery were significantly associated with greater reduction in triglycerides at follow up (β: 0.26 [95% CI: 0.11, 0.40], p = 0.001)—and, to a lesser degree, following fiber intervention (β: 0.09 [95% CI: 0.04, 0.15], p = 0.002). However, changes in isoUDCA were not associated with changes in body weight or glucose (Figure S5). Given the well-known effect of bariatric surgery on the modulation of satiety^30^^,^^31^ and the strong effect of bariatric surgery on isoUDCA levels, we have further investigated whether isoUDCA could be related to measures of satiety. We tested this by assessing the correlation between pre-breakfast reported appetite (a visual analog scale) in the ZOE PREDICT-1 study averaged over at least 3 breakfast meals (average 4.8) and fasting isoUDCA levels. We find a significant correlation between appetite levels pre-breakfast and isoUDCA (R^2^ = 0.047, F_(1,316)_ = 15.1, p = 4.8 × 10^−5^). This is shown in Figure 4D.

## Discussion

In this comprehensive analysis of serum and fecal BAs, we report that one of the secondary BAs studied, isoUDCA, is a marker of poor cardiometabolic health that is associated with higher fasting and post-prandial lipemia, inflammation, impaired liver function enzymes, and increased appetite scores. A classic twin heritability analysis indicates that fecal and serum levels of isoUDCA are moderately heritable, with most of the variance explained by environmental influences. Indeed, we report a strong contribution of the gut microbiome. We further show that circulating levels of isoUDCA significantly decreased post-bariatric surgery and modestly decreased following a 6-week dietary fiber intervention but are not modified by 6 weeks of omega-3 supplementation. Importantly, in healthy individuals, circulating levels of isoUDCA correlate with higher levels of pre-meal appetite. Bariatric surgery, the most effective and durable treatment for class III obesity,^32^ is known to result in decreased sensations of hunger and increased satiety and has been associated with enhanced delivery of primary BAs.^29^ However, unlike what has been reported for primary BAs, isoUDCA levels actually decreased after surgery, and given that its levels are ∼80% determined by gut microbes, this is further confirmation that some of the physiological effects of bariatric surgery are mediated by gut microbes.^33^ Our findings support that levels of the secondary BA isoUDCA may have causal links to hypertriglyceridaemia. Finally, we show that isoUDCA is involved in increased appetite, suggesting that a reduction in its levels may mediate increased satiety and improved lipemic control post-bariatric surgery.

isoUDCA is a steroid acid anion belonging to the dihydroxy BAs, alcohols, and derivatives class, which are compounds containing or derived from a BA or alcohol. It is an epimer of UDCA, a secondary BA produced in humans by intestinal bacteria that is also used in purified form as a pharmaceutical to treat biliary diseases such as primary biliary cholangitis and primary biliary cirrhosis.^34^ Approximately 25% of serum isoUDCA and 10% of serum UDCA are conjugated with either glucuronic acid or N-acetylglucosamine, indicating hepatic formation and systemic secretion of glycosidic conjugates.^35^ In cell-based assays, almost 50% of isoUDCA is metabolized to 3-oxo-UDCA within 4 h, making the exploration of its actions difficult to study.^36^ In our data, UDCA was not measured in serum and was detected in a small subset of stool samples (<19% in ZOE PREDICT-1). However, isoUDCA was seen in both types of biospecimens, suggesting that *in vivo*, in individuals not receiving oral doses of UDCA, UDCA is absorbed, excreted, utilized by the liver, or epimerized into isoUDCA. On the other hand, published data have shown that UDCA in a dose of 28–30 mg/kg/day increases risk of death and need for liver transplant by 2.3-fold among those with primary sclerosing cholangitis,^37^ indicating that UDCA itself at high circulating concentrations is linked to negative outcomes. Based on the above considerations, the association observed in our data between isoUDCA and various unfavorable health traits (e.g., higher post-prandial lipemia) cannot be interpreted as isoUDCA being directly involved, as this may reflect differences in the metabolism of UDCA.

The association between this secondary BA and fasting triglycerides has been previously observed.^38^ However, importantly, in this study, we also find a positive association with post-prandial lipemic response. This is an important discovery because post-prandial lipemic response has been associated with an increased risk of CVD and mortality^14^^,^^15^ and may provide stronger predictive value relative to fasting lipid values. As such, future research should assess isoUDCA as a measure of cardiovascular risk that may add value as a fasting measure.

Here, we also observe a positive association between serum levels of isoUDCA with the HSI, a calculated measure predictive of non-alcoholic fatty liver disease (NAFLD). We found that serum levels of isoUDCA were associated with increased levels of ALT and GGT but not aspartate aminotransferase (AST). This pattern of increased ALT and GGT without a significant increase in AST is a classic clinical presentation for NAFLD.^39^^,^^40^ Furthermore, serum levels of isoUDCA were also associated with post-prandial lipemia, which has been associated with liver fat deposition in other research.^12^ This suggests a role for isoUDCA in non-alcoholic fatty liver pathogenesis, although our study is limited because liver fat was not directly measured, and we do not have confirmation of the presence of NAFLD. Therefore, isoUDCA warrants further investigation in patients with confirmed NAFLD and possibly as a predictive measure prior to disease development.

In addition to links with clinical features, we identify the key microbiome composition features involved in isoUDCA levels. Using a machine-learning algorithm, we discovered that the gut microbiota was able to accurately predict isoUDCA levels in serum and stool. Likewise, we identified single gut microbial species that might be responsible for the increase or decrease of its circulating and fecal levels. For instance, we highlight the positive correlation between isoUDCA and 2 species belonging to the *Blautia* genus, namely *B. wexlerae* and *B. massilienses.* Previous evidence has shown that *Blautia* species have the capacity to transform primary BAs to secondary BAs through 7-α-dehydroxylation.^41^ On the other hand, Ocaña-Wilhelmi and colleagues^42^ did not detect a relationship between the overall *Blautia* genus and fecal abundances of isoUDCA in a small study of 16 individuals with class III obesity after bariatric surgery. However, only half (8) of the participants received the same type of bariatric surgery that was performed in our cohort. On the other hand, we identified negative correlations between isoUDCA in serum and stool and gut species known for contributing to short-chain fatty acid (SCFA) levels, such as *C. eutactus*^43^ and *M. smithii*.^44^^,^^45^ These findings are not surprising, as a potential crosstalk between SCFAs and BAs has been suggested.^46^ SCFAs and BAs cause opposing effects in the host health,^46^ with SCFAs being able to regulate lipid metabolism, inflammatory response, and appetite.^43^

Our findings provide insights into how our gut microbiota modulate our metabolism and the specificity for certain species to perform functions. Taken together, these suggest that isoUDCA is a modifiable risk factor. In fact, we found associations between dietary factors and isoUDCA levels, more specifically lower levels among those with a plant-oriented diet (measured by the plant diversity index). We also found reductions in isoUDCA after a fiber supplementation intervention (20 g daily of inulin for 6 weeks) and after bariatric surgery. Prior research has shown plant-oriented Mediterranean diets,^47^ inulin fiber supplementation,^48^ and bariatric surgery^49^ to reduce post-prandial lipemia. Of interest, prior research has also shown that a plant-oriented Mediterranean diet and bariatric surgery^50^ reduce liver fat in the context of NAFLD. Moreover, in a recent study,^51^ isoUDCA was the most strongly associated compound in the whole plasma metabolome with low gut microbiome α diversity, which is consistent with our results showing a very strong influence of the gut microbiome on levels of this compound and with it being linked to negative health traits.

The association between higher levels of serum isoUDCA and fasting appetite scores fits with studies from animal models showing that BAs can act as regulators of appetite hormones by activation of basolateral intestinal Takeda G-protein receptor 5.^52^ Changes in BAs with dietary fiber supplementation are also consistent with results from animal models showing that dietary fiber supplementation results in gut microbiome changes in the production of bile salt hydrolases.^53^ The potential for modification of isoUDCA to achieve metabolic benefits through diet, exercise, or other lifestyle measures warrants further in-depth investigation.

The current study is strengthened by a large well-characterized discovery cohort with twin data, a tightly controlled nutritional challenge with post-prandial responses measured, and two interventional studies that allowed us to explore changes in isoUDCA levels after dietary supplementation and bariatric surgery. Moreover, we include both men and women, from different countries, and clinical characteristics (e.g., healthy and class III obese), which make our results more generalizable.

### Limitations of the study

We also note some limitations. Firstly, the individuals included from ZOE PREDICT-1 are only females; however, we replicated the significant findings between isoUDCA and post-prandial lipemia in BARIA, which contains males and females. Secondly, post-surgical visits in BARIA are conducted 1 year post-surgery. During this time period, many other lifestyle changes may have occurred, including increased physical activity and improvements in dietary composition, which may represent unmeasured residual confounders. Moreover, these are self-reported and prone to various biases, including selective recall bias. Thirdly, BA levels were measured by Metabolon, which provides relative values and not absolute concentrations of the metabolites in serum or fecal water, so we cannot comment on the actual effect sizes. Additionally, the Metabolon platform was not able to curate serum UDCA because of interference from an unknown compound (X-24980) that elutes just before this analyte, and fecal abundances of UDCA were detected in <19% of the ZOE PREDICT-1 sample. Future studies should therefore disentangle the effect of UDCA from that of isoUDCA. Finally, from our study, we are unable to infer causality between isoUDCA levels and post-prandial lipemia and measures of liver function.

In conclusion, in this comprehensive assessment of serum and fecal BAs, using combined cohort studies, nutritional interventions, and surgical cohorts, we show that primary and secondary BAs have varied genetic heritability but that most are more powerfully predicted by unique environmental factors, like the gut microbiome and overall dietary pattern, rather than genetic factors. We identified a specific secondary BA, isoUDCA, that was associated with increased appetite, post-prandial lipemia, and post-prandial inflammation using either serum or stool testing. We also found serum isoUDCA associated with increased HSIs and liver inflammation tests. This suggests a prominent and previously undescribed role for isoUDCA in lipid metabolism, satiety signaling, and liver health. Serum isoUDCA levels were reduced by bariatric surgery and, more modestly, by inulin fiber supplementation, suggesting that levels of this marker for metabolic health can be modifiable through diet and other measures. This opens the door to future studies of isoUDCA as a measure and modifiable risk factor in lipid metabolism, CVD, and NAFLD.

## STAR★Methods

### Key resources table

**Table:** 

REAGENT or RESOURCE	SOURCE	IDENTIFIER
**Biological samples**		

Human serum bile acid metabolomics data	TwinsUK	Mendelary Data https://doi.org/10.17632/rp6fgdrn7d.1
	ZOE PREDICT-1	
	Omega-3 and fibre intervention study	
	BARIA	van Olden et al.^54^
Human faecal bile acid metabolomics data	TwinsUK	Mendelary Data https://doi.org/10.17632/8bfdytb3jx.1
	ZOE PREDICT-1	
Human faecal metagenomic data	TwinsUK	EBI (https://www.ebi.ac.uk/) accession number PRJEB32731
	ZOE PREDICT-1	EBI (https://www.ebi.ac.uk/) accession number PRJEB39223
Human serum postprandial triglycerides, glucose, insulin	ZOE-PREDICT-1	Berry et al.^13^
	BARIA	van Olden et al.^54^
Human serum liver function enzymes	TwinsUK	twinsuk.ac.uk/resources-for-researchers/access-our-data/

**Deposited data**		

Dietary data	TwinsUK	twinsuk.ac.uk/resources-for-researchers/access-our-data/
	ZOE PREDICT-1	

**Software and algorithms**		

R (version 4.0.2)	https://www.r-project.org/	N/A
Python using the numpy and scikit-learn packages		N/A
METs R package (version 1.2.7.1)	Comprehensive R Archive Network	cran.r-project.org/web/packages/mets/index.html
randomForest R package (version 4.7.1.1)	Comprehensive R Archive Network	cran.r-project.org/web/packages/randomForest/randomForest.pdf

### Resource availability

#### Lead contact

Further information and requests for resources and reagents should be directed to and will be fulfilled by the lead contact, Cristina Menni (cristina.menni@kcl.ac.uk).

#### Materials availability

This study did not generate new unique materials/reagents.

### Experimental model and subject details

#### Discovery cohorts

TwinsUK^55^: We included 2,382 individuals with serum and stool primary and secondary bile acids measured by Metabolon inc. using non-targeted metabolomic profiling, along with shotgun metagenomes and dietary information. All twins provided informed written consent and the study was approved by St. Thomas’ Hospital Research Ethics Committee (REC Ref: EC04/015).

ZOE PREDICT-1^13^: We also included 327 females from the UK based ZOE PREDICT-1 study (IRAS 236407) - in a post hoc analyses, with serum and stool bile acids measured by Metabolon inc., shotgun metagenomes, postprandial markers, liver function and who completed a food frequency questionnaire (FFQ).The ZOE PREDICT-1 study,^13^ was a single-arm nutritional intervention conducted between June 2018 and May 2019. Participants attended a full day clinical visit consisting of test meal challenges followed by a 13-day home-based phase.^13^ During the clinical visit, blood samples were taken at specific timepoints and increments as previously described,^13^ from which blood triglyceride, glucose, insulin, and glycoprotein acetylation (GlycA) (as a marker of inflammation).^13^ GlycA is a proton nuclear magnetic resonance spectroscopy signal that reflects the methyl groups bound to N-acetylglucosamine residues attached to circulating plasma proteins and is recognised and validated as a biomarker of systemic inflammation.^56^ For each of these variables, we considered (i) the baseline fasting measures; (ii) the peak (over the 6-h visit for triglycerides and GlycA, and 2 hours for insulin and glucose)^13^ and (iii) the magnitude of increase (delta increase = peak - baseline). Ethical approval for ZOE PREDICT-1 was obtained from St. Thomas Hospital research ethics committees. All individuals provided informed written consent (IRAS 236407) and the trial was registered on ClinicalTrials.gov (registration number: NCT03479866).

#### Replication cohorts

BARIA^54^: BARIA is a longitudinal Dutch cohort of obese individuals scheduled to undergo bariatric surgery (94% laparoscopic Roux-en-Y gastric bypass (LRYGB)) designed to identify gut microbial, immunological, and metabolic markers that can influence the pathways involved in the pathogenesis of obesity, type 2 diabetes, and non-alcoholic fatty liver disease. Preoperative data collection includes demographic, biometric, and blood measurements (from which triglyceride levels are measured). Participants also consume a mixed meal challenge (MMT) (74.3g carbohydrates, 23.3g fat, and 24g protein), with further blood sampling taken 10, 20, 30, 60, 90 and 120 minutes thereafter. In a repeated measure study protocol, participants are re-characterised 1-year (±1 month) post-surgery. BARIA received ethical approval from the Ethical Review Board of the Academic Medical Center, Amsterdam (NL55755.018.15).

The Omega-3 and fibre intervention study^26^: We included 65 participants from TwinsUK who underwent a 6-week randomised intervention trial designed to explore the influence of dietary Omega-3 or fibre supplementation on gut microbial composition, as previously described.^26^ Participants were randomised to one of two intervention arms, the first arm were administered 20g of oral inulin fibre, while the second arm were given 500mg of omega-3 supplements (165mg of EPA, 110mg DHA in gelatin capsules) daily for 6 weeks. The trial was approved by the West Midlands Black Country Research Ethics Committee (18/WM/0066) and registered under the clinicaltrials.gov database (NCT03442348). Further details are included in the Supplementary methods.

#### Ethics

All participants provided written informed consent. TwinsUK BioBank was approved by NHS North West - Liverpool Central Research Ethics Committee (REC reference 19/NW/0187), IRAS ID 258513. This approval supersedes earlier approvals granted to TwinsUK by St Thomas Hospital Research Ethics Committee (REC reference EC04/015), which have now been subsumed within the TwinsUK BioBank. Ethical approval for the PREDICT study was obtained from the NHS London - Hampstead Research Ethics Committee (REC reference 18/LO/0663) and all individuals provided informed written consent, and the trial was registered on ClinicalTrials.gov (registration number: NCT03479866). The Omega-3 and fibre intervention study was approved by the West Midlands Black Country Research Ethics Committee (18/WM/0066) and registered under the clinicaltrials.gov database (registration number: NCT03442348). BARIA received ethical approval from the Ethical Review Board of the Academic Medical Center, Amsterdam (NL55755.018.15).

### Method details

#### Bile acids metabolomics profiling

Bile acid concentrations were measured from faecal and serum samples by Metabolon Inc. (Durham, USA) using an untargeted LC-MS platform. All samples were maintained at −80°C until processing. Briefly, following precipitation in methanol and centrifuging (Glen Mills GenoGrinder 2000) the resulting extract was divided into five fractions; both aliquots (1) and (2) were analysed using acidic positive ion conditions and chromatographically optimised for hydrophilic and hydrophobic compounds respectively, aliquot (3) was analysed using a basic negative ion optimised conditions using a dedicated separate dedicated C18 column, aliquot (4) was analysed using negative ionisation following elution from a hydrophilic interaction liquid chromatography column, while aliquot (5) was reserved as a backup.

Several controls were analysed in concert with experimental samples. (i) extracted water samples served as process blanks; (ii) and a cocktail of standards, known not to interfere with measurements, spiked into every analysed sample facilitated instrument performance monitoring and aided chromatographic alignment. Instrument variability was determined by calculating the median relative standard deviation (RSD) for the standards that were added to each sample prior to injection into the mass spectrometers. Overall process variability was determined by calculating the median RSD for all endogenous metabolites (i.e., non-instrument standards) present in 100% or more of the pooled technical replicate samples. Experimental samples and controls were randomised across the platform run.

#### Compound identification

Metabolites were identified by comparison of the ion features in the experimental samples to a reference library of chemical standard entries that included retention time/index, molecular weight (m/z), and MS spectra. Identification of known chemical entities is based on comparison across all 3 features to metabolomic library entries of purified standards. More than 3300 commercially available purified standard compounds have been acquired and registered into the library, while additional mass spectral entries have been created for structurally unnamed biochemicals, which have been identified by virtue of their recurrent nature (both chromatographic and mass spectral). These compounds have the potential to be identified by future acquisition of a matching purified standard or by classical structural analysis.

#### Metabolite quantification and normalisation

Peaks were quantified using area-under-the-curve. Raw area counts for each metabolite in each sample were normalised to correct for variation resulting from instrument inter-day tuning differences by the median value for each run-day, therefore, setting the medians to 1.0 for each run. This preserved variation between samples but allowed metabolites of widely different raw peak areas to be compared on a similar graphical scale.

#### QC

To remove batch variability from bile acid runs, for each bile acid, the values in the experimental samples were divided by the median of those samples in each instrument batch, giving each batch and thus the bile acid a median of one. Missing values were imputed, using the minimum value across all batches in the median scaled data.

From metabolomic profiling, 26 bile acids were detected in serum, of which 10 primary and 16 secondary, while 43 bile acids in stool (10 primary and 33 secondary). Bile acids with more than 20% missingness were excluded. Bile acids were batch normalised, missing values imputed and inverse normalised. After cleaning 19 bile acids in serum (8 primary and 11 secondary) and 15 bile acids in stool (3 primary and 12 secondary) remained. A total of 4 bile acids overlapped between serum and faecal samples (Table S3).

#### Dietary information

Habitual dietary information was estimated via a 131-item modified European Prospective Investigation into Cancer and Nutrition food frequency questionnaire (FFQ), as previously described.^57^ From which, we calculated indexes to represent whole dietary patterns, including the healthy eating index, which characterizes intakes of foods and nutrients understood to associate with chronic diseases,^58^ and the original plant diversity index (oPDI), as a measure of plant food intakes compared to animal foods,^24^ the alternative Mediterranean diet index, and the healthy eating index.

#### Microbiome sequencing and profiling

TwinsUK and ZOE PREDICT-1 participants collected stool samples at home in pre-labelled kits (containing 2 x 25ml tube or 1 x 25ml tube and 1 x 10ml Zymo buffer) posted to them prior to their clinic visit date and brought with them to the visit. Alternatively, samples can be posted to the clinic using blue Royal Mail safe boxes. In the laboratory, samples were homogenised, aliquoted into 4 bijou tubes, and stored at −80 °C, within 2 hours of receipt. Deep shotgun metagenomic sequencing in serum and stool samples from TwinsUK and ZOE PREDICT-1 was performed as previously described.^59^^,^^60^

#### Appetite

Appetite levels before each standardised meal in the ZOE PREDICT-1 study was self-declared by participants in the period +/-5 minutes around the start of consumption of the meal. Readings are self-reported on a scale of 0-100. Larger values indicate increased perception of appetite (see^61^). The levels before each standardised meal (min 0) were collected and the values used to assess for correlation with serum levels of bile acids correspond to the average of 4.8 (SD=0.53) breakfasts as part of the “at home” phase of the ZOE PREDICT-1 study.

### Quantification and statistical analysis

Statistical analysis was carried out using R, version 4.0.2, and python, including the libraries, Numpy, scikit-learn.

#### Heritability

Taking advantage of the twin nature of our data, we estimated the heritability of serum and stool BA concentrations using structural equation modelling. Heritability modelling decomposes the observed phenotypic variance into three latent sources of variation: additive genetic variance (A), shared/common environmental variance (C) and non-shared/unique environmental variance (E).^62^ Additive genetic influences are indicated when monozygotic (MZ) twins are more similar than dizygotic (DZ) twins. The common environmental component estimates the contribution of the family environment which is assumed to be equal in both MZ and DZ twin pairs, whereas the unique environmental component does not contribute to twin similarity, rather it estimates the effects that apply only to each individual and includes measurement error.^63^ Any greater similarity between MZ twins than DZ twins is attributed to greater sharing of genetic influences. Heritability is defined as the proportion of the phenotypic variation attributable to genetic factors. The Akaike information criterion (AIC) was used to determine the best-fitting model (comparing the saturated model to the ACE, AE and CE models). The model with the lowest AIC reflects the best balance of goodness of fit and parsimony.^62^ Heritability estimates were computed by pulling together the TwinsUK and ZOE PREDICT-1 cohort using the package METs (version 1.2.7.1)^64^ in R (version 4.0.2).

#### Gut microbiota contribution to BA levels

To quantify to which extent the gut microbiota is able to predict faecal and serum BA levels, we used Random Forest (RF) models as they have been repeatedly shown to be particularly suitable and robust to the statistical challenges inherent to microbiome data.^65^ Results were replicated in the ZOE PREDICT-1 cohort by re-running the models. RF was performed using the randomForest function in R, regressors (ntree=1000 and mtry= a third of features number) and classifiers (ntree=1000 and mtry=square root of features number) with compositional data using 5-folds cross-validation. Before running the models, predictors with variance zero or near to zero were excluded, and to avoid overfitting, for TwinsUK, any twin from the training fold was removed if their twin was present in the test fold. For the regressors, performance was calculated using the mean of the Spearman's correlations between the real and predicted BA levels over the 5 folds used as a test set. For the classifiers, the continuous response was converted into two classes based on the top and bottom quartiles, and their performance was evaluated using the mean of the obtained area under the receiver operating characteristic curve (AUC) values over the 5 folds.

The influence of single gut microbial species on isoUDCA circulating and faecal levels was then investigated using partial Spearman’s correlations adjusted for age, BMI, and sex assigned at birth in TwinsUK and validated in ZOE PREDICT-1. Only species with a prevalence of >20% were included in the analysis. P-values were corrected for multiple testing using FDR through the Benjamini and Hochberg method (FDR<0.05) for TwinsUK and a nominal p-value<0.05 for ZOE PREDICT-1. The characterised species that were significantly correlated with isoUDCA in serum and stool and presented the same directional effects in TwinsUK and ZOE PREDICT-1, were represented separately for each cohort, using the Euclidean distance for clustering the heatmap with the complete linkage method.

#### Association between BAs and post-prandial lipaemia, inflammation, liver function and diet

We first used linear regression models to evaluate the associations between serum and faecal BA and postprandial lipaemia, adjusting for age, BMI and multiple testing using FDR (q<0.05). We then further leveraged linear models adjusted for age and BMI to explore the relationship between serum and faecal levels of the secondary bile acid isoUDCA and; (i) liver function biomarkers; (ii) postprandial parameters; (iii) inflammation as determined by glycoprotein acetylation (GlycA) measured at specific timepoints and increments.^13^ GlycA is a proton nuclear magnetic resonance spectroscopy signal that reflects the methyl groups bound to N-acetylglucosamine residues attached to circulating plasma proteins and is recognised and validated as a biomarker of systemic inflammation,^56^ and (iv) dietary intake (nutrients, foods and dietary patterns). We additionally conducted a sensitivity analysis by further adjusting for total fat intake as a percentage of total energy intake.

Linear regression models were also employed to replicate the positive association between fasting isoUDCA levels and post-prandial lipaemia in the pre-surgery BARIA cohort adjusting for age, sex assigned at birth, BMI and T2D status.

#### Change in isoUDCA levels after dietary intervention and post bariatric surgery

Lastly, to explore changes in isoUDCA levels post bariatric surgery, and post dietary intervention we used paired t.tests.

## Data Availability

The data used in this study are held by the Department of Twin Research at King’s College London. The data can be released to bona fide researchers using our normal procedures overseen by the Wellcome Trust and its guidelines as part of our core funding (https://twinsuk.ac.uk/resources-for-researchers/access-our-data/). The gut microbiome data is available on EBI (https://www.ebi.ac.uk/) under accession number PRJEB39223 (ZOE- PREDICT-1) and PRJEB32731 (TwinsUK). Bile acid data is available at Mendeley data: https://doi.org/10.17632/rp6fgdrn7d.1 and https://doi.org/10.17632/8bfdytb3jx.1. The code, metabolomics, and any additional information required to reanalyze the data reported in this paper is available from the lead contact upon request.

## Supplemental information

Document S1. Figures S1–S5 and Tables S1–S4

Document S2. Article plus supplemental information
