# Supplementary material for: The secondary bile acid isoursodeoxycholate correlates with post-prandial lipemia, inflammation, and appetite and changes post-bariatric surgery
Source: Cell Rep Med. 2023 Apr 5;4(4):100993. doi: 10.1016/j.xcrm.2023.100993 (PMC10140478; doi:10.1016/j.xcrm.2023.100993)
Supplement: Document S1. Figures S1–S5 and Tables S1–S4 [file mmc1.pdf]

Cell Reports Medicine, Volume *4*

Supplemental information

The secondary bile acid isoursodeoxycholate correlates with post-prandial lipemia, inflammation, and appetite and changes post-bariatric surgery

Panayiotis Louca, Abraham S. Meijnikman, Ana Nogal, Francesco Asnicar, Ilias Attaye, Amrita Vijay, Afroditi Kouraki, Alessia Visconti, Kari Wong, Sarah E. Berry, Emily R. Leeming, Olatz Mompeo, Francesca Tettamanzi, Andrei-Florin Baleanu, Mario Falchi, George Hadjigeorgiou, Jonathan Wolf, Yair I.Z. Acherman, Arnold W. Van de Laar, Victor E.A. Gerdes, Gregory A. Michelotti, Paul W. Franks, Nicola Segata, Massimo Mangino, Tim D. Spector, William J. Bulsiewicz, Max Nieuwdorp, Ana M. Valdes, and Cristina Menni

The secondary bile acid isoursodeoxycholate (isoUDCA) modulates postprandial lipaemia, inflammation and appetite, and changes post bariatric surgery

Panayiotis Louca[1], Abraham S. Meijnikman[2], Ana Nogal[1], Francesco Asnicar[3], Ilias Attaye[2], Amrita Vijay[4,5], Afroditi Kouraki[4,5], Alessia Visconti[1], Kari Wong[6], Sarah E. Berry[7], Emily R. Leeming[1], Olatz Mompeo[1], Francesca Tettamanzi[1], Andrei-Florin Baleanu[1], Mario Falchi[1], George Hadjigeorgiou[8], Jonathan Wolf[8], Yair I. Z. Acherman[9], Arnold W. Van de Laar[9], Victor E.A. Gerdes[2], Gregory A. Michelotti[6], Paul W. Franks[10,11], Nicola Segata[3], Massimo Mangino[1,12], Tim D. Spector[1], William J. Bulsiewicz[8], Max Nieuwdorp[2], Ana M. Valdes[4,5,13,*], Cristina Menni[1,13,*]

Supplementary tables, and figures

Supplementary Table 1. Descriptive characteristics of participants **2**

Supplementary Table 2. Bile acids detected in >80% of the serum and stool samples. **4**

Supplementary Table 3. Bile acids correlations with the gut microbiome **5**

Supplementary Table 4. Correlation of primary and secondary bile acids measured from serum and stool samples and postprandial lipaemia in PREDICT. **8**

Supplementary Figure 1. Correlation between concentrations of bile acids in serum and stool in TwinsUK and ZOE PREDICT-1. Colours represent Spearman's rho values, x signifies a p value > 0.05. **10**

Supplementary Figure 2. Line plot of serum bile acid concentrations at fasting and in response to test meal challenge in ZOE PREDICT-1. Each grey line represents an individual participant, the blue line the mean and the red line the median. **11**

Supplementary Figure 3. Correlation of primary and secondary bile acids measured from serum and stool samples and postprandial lipaemia in PREDICT. * denotes bile acids that passed multiple testing (FDR <0.05). Bars with thick black borders represent bile acids detected in both serum and stool samples. **12**

Supplementary Figure 4. Partial Spearman's correlations adjusted for age and BMI between isoUDCA levels in serum and stool, and single microbial species in 327 women from ZOE PREDICT-1. Only the characterised species with a prevalence >20% that had significant correlations (p-value<0.05), and presented the same directional effects in TwinsUK and ZOE PREDICT-1 are shown. Species are hierarchically clustered (complete linkage, Euclidean distance). **13**

Supplementary Figure 5. Correlation between change in isoUDCA and changes in triglycerides, body weight, and glucose, in the BARIA trial and in the fibre arm of the Omega-3 and Fibre Intervention Study to Improve Metabolic Health study. Points represent regression coefficients (solid if statistically significant) adjusting for age, and BMI, and tails illustrate 95% confidence intervals. **14**

Supplementary Table 1. Descriptive characteristics of participants

	TwinsUK (n = 2,382)		ZOE PREDICT-1 (n = 327)		Omega-3 Study (n = 65)		BARIA (n = 116)			
							Pre-surgery (n = 116)		*Post-surgery (n = 71)*	
	**n**	**%**	**n**	**%**	**n**	**%**	**n**	**%**	**n**	**%**
Females	2,037	85.5	327	100	61	93.9	91	78.5	68	95.8
Type-2 diabetes	67	2.8	0	0	0	0	26	28.6	19	26.8
	mean	***sd***	***mean***	***sd***	***mean***	***sd***	***mean***	***sd***	***Mean***	***sd***
Age, years	58.9	14.6	53.8	7.0	65.9	9.4	46.0	10.3	47	9.6
BMI, kg/m²	26.0	5.0	26.3	5.6	26.5	4.4	39.6	4.5	26.0	2.9
Fasting										
Triglycerides	1.0	0.6	1.1	0.57			1.3	0.7		
GlycA			1.35	0.19						
Postprandial										
Triglycerides			2.33	1.2			1.5	0.7		
GlycA			1.4	0.3						
Dietary indices										
HEI	61.8	9.9	57.1	9.4						

aMED	4.8	1.9	4.5	1.8
oPDI	56.1	5.6	53.3	6.1
hPDI	58.5	6.7	58.9	6.2
uPDI	58.9	7.0	59.4	6.2

Supplementary Table 2. Bile acids detected in >80% of the serum and stool samples.

Bile acid	Pathway	Serum	Stool
**Cholate**	Primary	Yes	Yes
**Glycocholate**	Primary	Yes	Yes
**Glycochenodeoxycholate**	Primary	Yes	Yes
**Taurocholate**	Primary	Yes	No
**Glyco-beta-muricholate**	Primary	Yes	No
**Glycochenodeoxycholate 3-sulfate**	Primary	Yes	No
**Glycochenodeoxycholate glucuronid**	Primary	Yes	No
**Taurochenodeoxycholate**	Primary	Yes	No
**Isoursodeoxycholate**	Secondary	Yes	Yes
**Deoxycholic acid 12 sulfate**	Secondary	Yes	No
**Deoxycholic acid glucuronide**	Secondary	Yes	No
**Glycocholenate sulfate**	Secondary	Yes	No
**Glycodeoxycholate 3 sulfate**	Secondary	Yes	No
**Glycolithocholate sulfate**	Secondary	Yes	No
**Glycoursodeoxycholate**	Secondary	Yes	No
**Glycoursodeoxycholicacid sulfate 1**	Secondary	Yes	No
**Lithocholatesulfate1**	Secondary	Yes	No
**Taurocholenatesulfate**	Secondary	Yes	No
**Taurolithocholate 3 sulfate**	Secondary	Yes	No
**Dehydrodeoxycholate**	Secondary	No	Yes
**Dehydrolithocholate**	Secondary	No	Yes
**Deoxycholate**	Secondary	No	Yes
**Glycodeoxycholate**	Secondary	No	Yes
**Hyocholate**	Secondary	No	Yes
**Isohyodeoxycholate**	Secondary	No	Yes
**Ketodeoxycholate**	Secondary	No	Yes
**Ketolithocholate**	Secondary	No	Yes
**Lithocholate**	Secondary	No	Yes
**Oxolithocholate**	Secondary	No	Yes
**Ursocholate**	Secondary	No	Yes

Supplementary Table 3. Bile acids correlations with the gut microbiome

	TwinsUK								ZOE PREDICT-1							
	**Serum**				**Stool**				**Serum**				**Stool**			
**Bile acid**	**AUC (%)**	**95% CI**	**Rho**	**95% CI**	**AUC (%)**	**95% CI**	**Rho**	**95% CI**	**AUC (%)**	**95% CI**	**Rho**	**95% CI**	**AUC (%)**	**95% CI**	**Rho**	**95% CI**
**Cholate**	80.7	80.5, 80.9	0.42	0.42, 0.42	83.4	83.3, 83.5	0.48	0.48, 0.48	80.9	79.8, 82.0	0.43	0.42, 0.44	84.1	83.1, 85.2	0.46	0.45, 0.47
**glycocholate**	49.2	48.6, 49.8	0.06	0.06, 0.06	72.9	72.5, 73.4	0.35	0.35, 0.35	56.9	55.5, 58.3	0.04	0.03, 0.05	80.7	79.6, 81.9	0.40	0.39, 0.41
**glycochenodeoxycholate**	60.3	59.9, 60.6	0.12	0.12, 0.12	75.2	74.7, 75.6	0.37	0.37, 0.37	63.1	61.9, 64.4	0.23	0.22, 0.24	79.2	79.6, 80.4	0.42	0.41, 0.43
**isoursodeoxycholate**	75.4	75.0, 75.8	0.39	0.39, 0.39	85.2	84.8, 85.6	0.51	0.50, 0.52	85.0,	84.4, 86.0	0.47	0.45, 0.49	87.3	87.3, 87.8	0.48	0.47, 0.49
**deoxycholic acid 12-sulfate**	69.3	68.8, 69.9	0.27	0.27, 0.27					79.4	78.7, 80.2	0.43	0.42, 0.44				
**deoxycholic acid glucuronide**	68.8	68.4, 69.3	0.34	0.33, 0.35					60.2	60.2, 61.4	0.16	0.15, 0.17				
**glyco-beta-muricholate**	61.3	61.1, 61.5	0.20	0.19, 0.21					70.4	68.2, 72.7	0.16	0.15, 0.17				
**glycochenodeoxycholate 3-sulfate**	51.4	50.9, 52.0	0.09	0.09, 0.09					58.4	57.7, 59.1	0.19	0.18, 0.20				

**glycochenodeoxycholate glucuronide**	55.6	55.2, 56.1	0.09	0.09, 0.09					59.9	58.6, 61.3	0.24	0.23, 0.25				
**glycocholenate sulfate**	60.8	60.3, 61.3	0.10	0.10, 0.10					61.4	60.1, 62.6	0.09	0.08, 0.10				
**glycodeoxycholate 3-sulfate**	66.8	66.6, 67.1	0.21	0.21, 0.21					69.1	67.5, 70.7	0.25	0.24, 0.26				
**glycolithocholate sulfate**	66.6	66.1, 67.2	0.28	0.28, 0.28					71.9	70.3, 73.4	0.32	0.31, 0.33				
**glycoursodeoxycholate**	75.8	75.3, 76.3	0.40	0.40, 0.40					73.0	72.2, 73.9	0.33	0.32, 0.34				
**glycoursodeoxycholic acid sulfate**	79.0	78.4, 79.6	0.42	0.42, 0.42					65.7	63.0, 68.3	0.27	0.26, 0.28				
**lithocholate sulfate**	72.0	71.6, 72.5	0.30	0.30, 0.30					71.3	70.0, 72.8	0.32	0.31, 0.33				
**taurochenodeoxycholate**	61.8	61.3, 62.3	0.11	0.11, 0.11					53.6	53.0, 54.2	0.14	0.12, 0.16				
**taurocholate**	52.0	51.4, 52.7	0.01	0, 0.02					52.1	51.4, 52.9	0.06	0.05, 0.07				
**taurocholenate sulfate**	62.2	61.5, 62.9	0.14	0.14, 0.14					60.6	59.2, 61.9	0.11	0.10, 0.12				
**taurolithocholate 3-sulfate**	72.3	71.7, 72.9	0.33	0.33, 0.33					71.6	70.6, 72.7	0.33	0.32, 0.34				
**12-ketolithocholate**					72.0	71.7, 72.4	0.33	0.33, 0.33					81.2	80.3, 82.2	0.40	0.39, 0.41

**3-dehydrodeoxycholate**	71.8	71.3, 72.3	0.36	0.36, 0.36	84.0	82.8, 85.2	0.51	0.50, 0.52
**6-oxolithocholate**	67.3	66.8, 67.8	0.23	0.22, 0.24	71.6	70.8, 72.3	0.29	0.28, 0.30
**7-ketodeoxycholate**	82.4	82.1, 82.1	0.46	0.45, 0.47	83.0	82.3, 83.7	0.45	0.44, 0.46
**dehydrolithocholate**	81.7	81.5, 82.0	0.47	0.47, 0.47	79.6	78.5, 80.8	0.48	0.46, 0.50
**Deoxycholate**	83.7	83.5, 84.0	0.48	0.48, 0.48	86.5	85.8, 87.2	0.51	0.50, 0.52
**glycodeoxycholate**	76.1	75.7, 76.5	0.37	0.37, 0.37	76.6	75.1, 78.2	0.36	0.35, 0.37
**hyocholate**	80.5	80.1, 80.9	0.43	0.42, 0.44	78.8	78.8, 79.9	0.40	0.39, 0.41
**isohyodeoxycholate**	74.4	73.5, 75.2	0.35	0.35, 0.35	58.1	56.6, 59.6	0.04	0.03, 0.05
**lithocholate**	75.9	75.3, 76.5	0.38	0.38, 0.38	75.2	74.1, 76.2	0.39	0.38, 0.4
**ursocholate**	82.1	81.5, 82.8	0.50	0.50, 0.50	83.7	82.8, 84.6	0.47	0.46, 0.48

Supplementary Table 4. Correlation of primary and secondary bile acids measured from serum and stool samples and postprandial lipaemia in PREDICT.

		Serum			Stool		
**Bile acid**	**Pathway**	**Beta**	**95% CI**	**p value**	**Beta**	**95% CI**	**p value**
**Cholate**	Primary	-0.04	-0.17, 0.1	0.59	0.12	-0.02, 0.26	0.09
**Glycocholate**	Primary	0.1	-0.06, 0.26	0.21	0.01	-0.12, 0.14	0.89
**Glycochenodeoxycholate**	Primary	0.12	-0.05, 0.29	0.18	0.06	-0.09, 0.21	0.41
**Taurocholate**	Primary	0.06	-0.10, 0.21	0.46	NA	NA	NA
**Glyco-beta-muricholate**	Primary	0.03	-0.13, 0.18	0.73	NA	NA	NA
**Glycochenodeoxycholate 3-sulfate**	Primary	-0.05	-0.19, 0.09	0.5	NA	NA	NA
**Glycochenodeoxycholate glucuronide**	Primary	0.12	-0.02, 0.27	0.1	NA	NA	NA
**Taurochenodeoxycholate**	Primary	0.02	-0.15, 0.19	0.8	NA	NA	NA
**Isoursodeoxycholate**	Secondary	0.32	0.18, 0.46	7.36 × 10-6	0.23	0.10, 0.36	5.52 × 10-4
**Deoxycholic acid 12-sulfate**	Secondary	0.14	-0.01, 0.30	0.06	NA	NA	NA
**Deoxycholic acid glucuronide**	Secondary	0.17	0.02, 0.32	0.02	NA	NA	NA
**Glycocholenate sulfate**	Secondary	0.03	-0.10, 0.16	0.65	NA	NA	NA
**Glycodeoxycholate 3-sulfate**	Secondary	-35.6	-0.15, 0.14	0.97	NA	NA	NA

**Glycolithocholate sulfate**	Secondary	5 × 10-3	-0.13, 0.14	0.94	NA	NA	NA
**Glycoursodeoxycholate**	Secondary	0.26	0.10, 0.42	1.6 × 10-3	NA	NA	NA
**Glycoursodeoxycholic acid sulfate**	Secondary	0.18	0.03, 0.34	0.02	NA	NA	NA
**Lithocholate sulfate**	Secondary	0.01	-0.14, 0.15	0.94	NA	NA	NA
**Taurocholenate sulfate**	Secondary	-0.12	-0.27, 0.03	0.12	NA	NA	NA
**Taurolithocholate 3-sulfate**	Secondary	-0.09	-0.22, 0.05	0.22	NA	NA	NA
**3-Dehydrodeoxycholate**	Secondary	NA	NA	NA	0.19	0.06, 0.31	4.47 × 10-3
**Dehydrolithocholate**	Secondary	NA	NA	NA	0.04	-0.10, 0.17	0.6
**Deoxycholate**	Secondary	NA	NA	NA	0.21	0.08, 0.33	1.10 × 10-3
**Glycodeoxycholate**	Secondary	NA	NA	NA	0.13	-0.02, 0.28	0.09
**Hyocholate**	Secondary	NA	NA	NA	0.2	0.06, 0.34	0.01
**Isohyodeoxycholate**	Secondary	NA	NA	NA	0.12	-0.02, 0.26	0.1
**7-Ketodeoxycholate**	Secondary	NA	NA	NA	0.12	-0.02, 0.26	0.1
**12-Ketolithocholate**	Secondary	NA	NA	NA	0.15	0.01, 0.28	0.03
**Lithocholate**	Secondary	NA	NA	NA	0.13	0.00, 0.26	0.05
**6-Oxolithocholate**	Secondary	NA	NA	NA	4.45 × 10-3	-0.12, 0.13	0.95
**Ursocholate**	Secondary	NA	NA	NA	0.26	0.12, 0.39	1.86 × 10-4

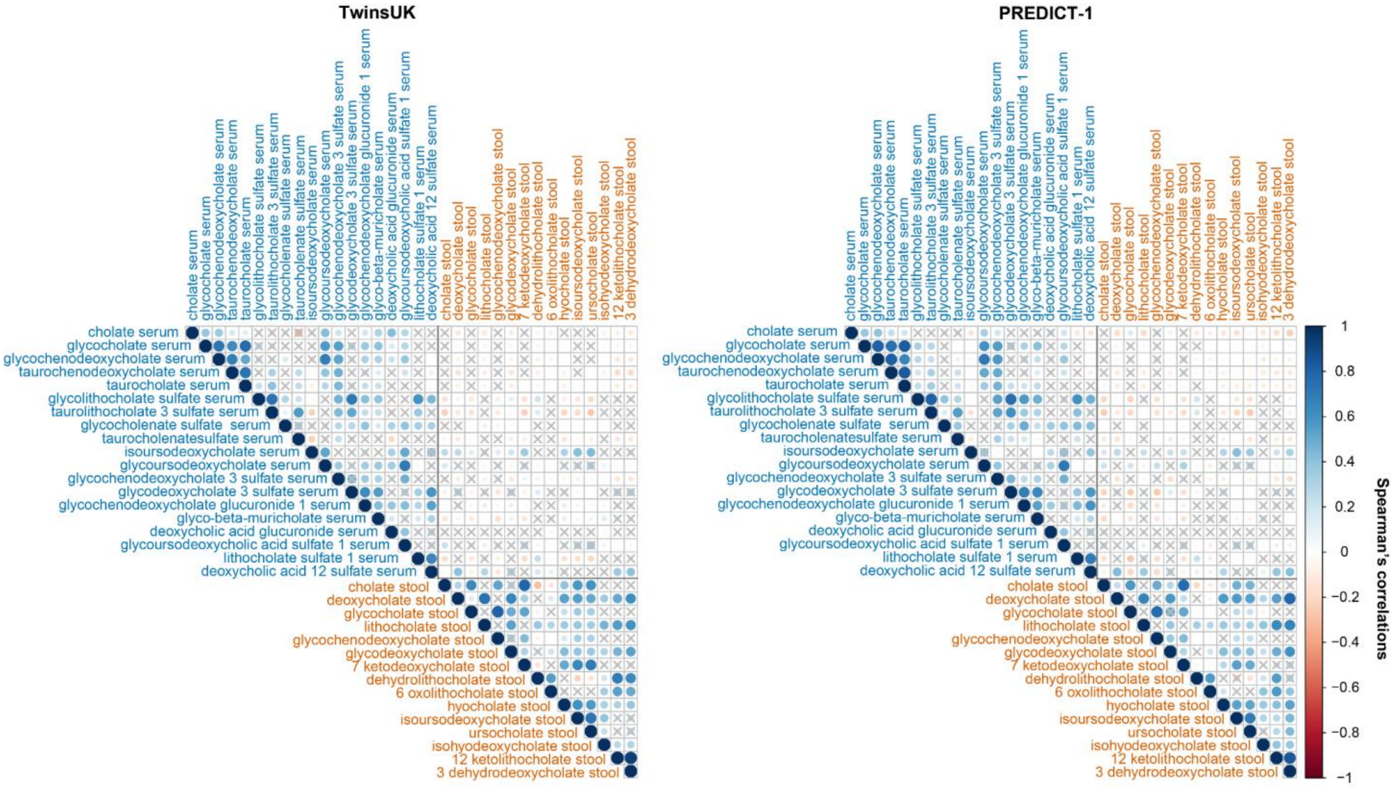

Supplementary Figure 1. Correlation between concentrations of bile acids in serum and stool in TwinsUK and ZOE PREDICT-1. Colours represent Spearman's rho values, x signifies a p value > 0.05.

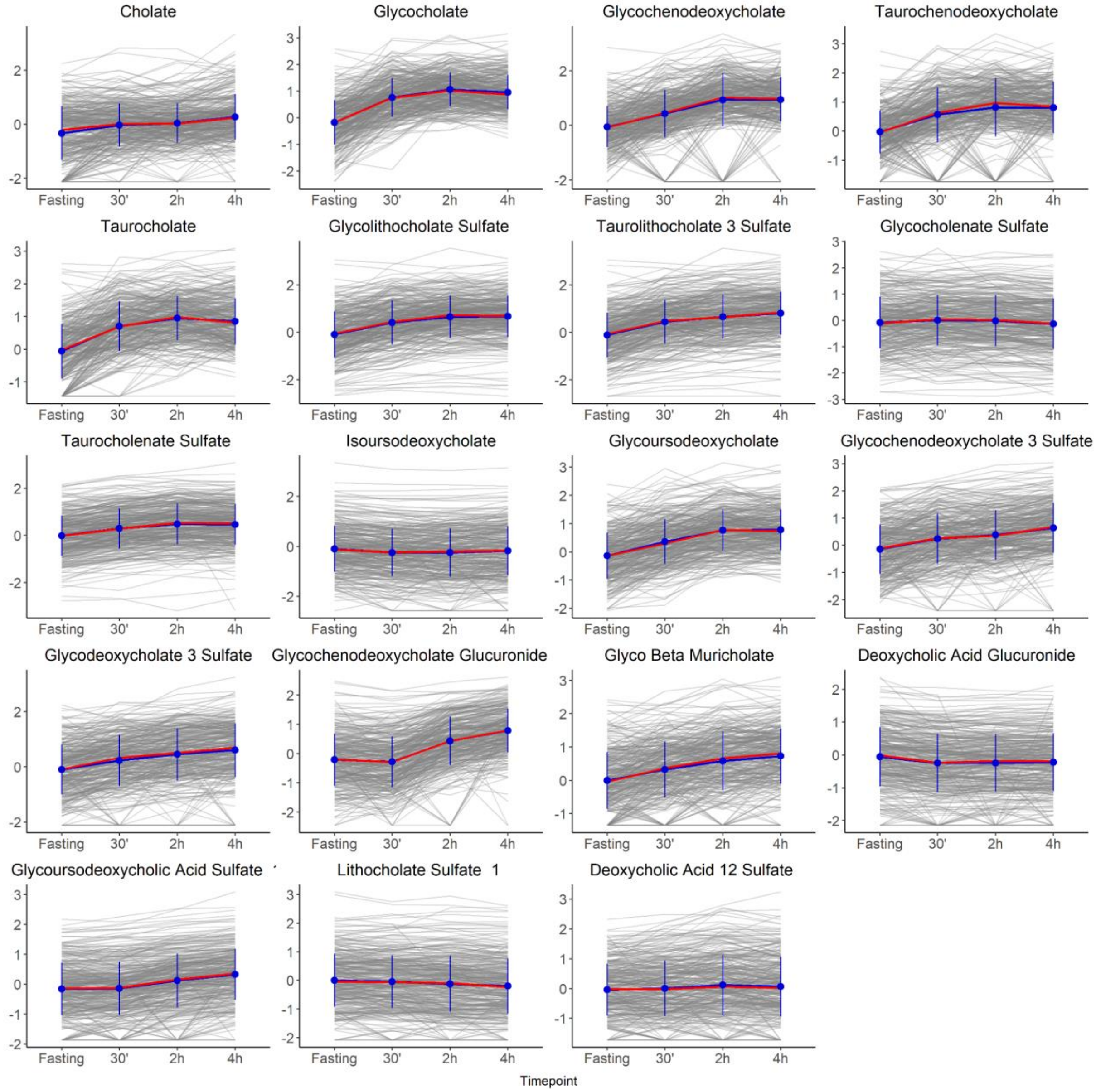

Supplementary Figure 2. Line plot of serum bile acid concentrations at fasting and in response to test meal challenge in ZOE PREDICT-1. Each grey line represents an individual participant, the blue line the mean and the red line the median.

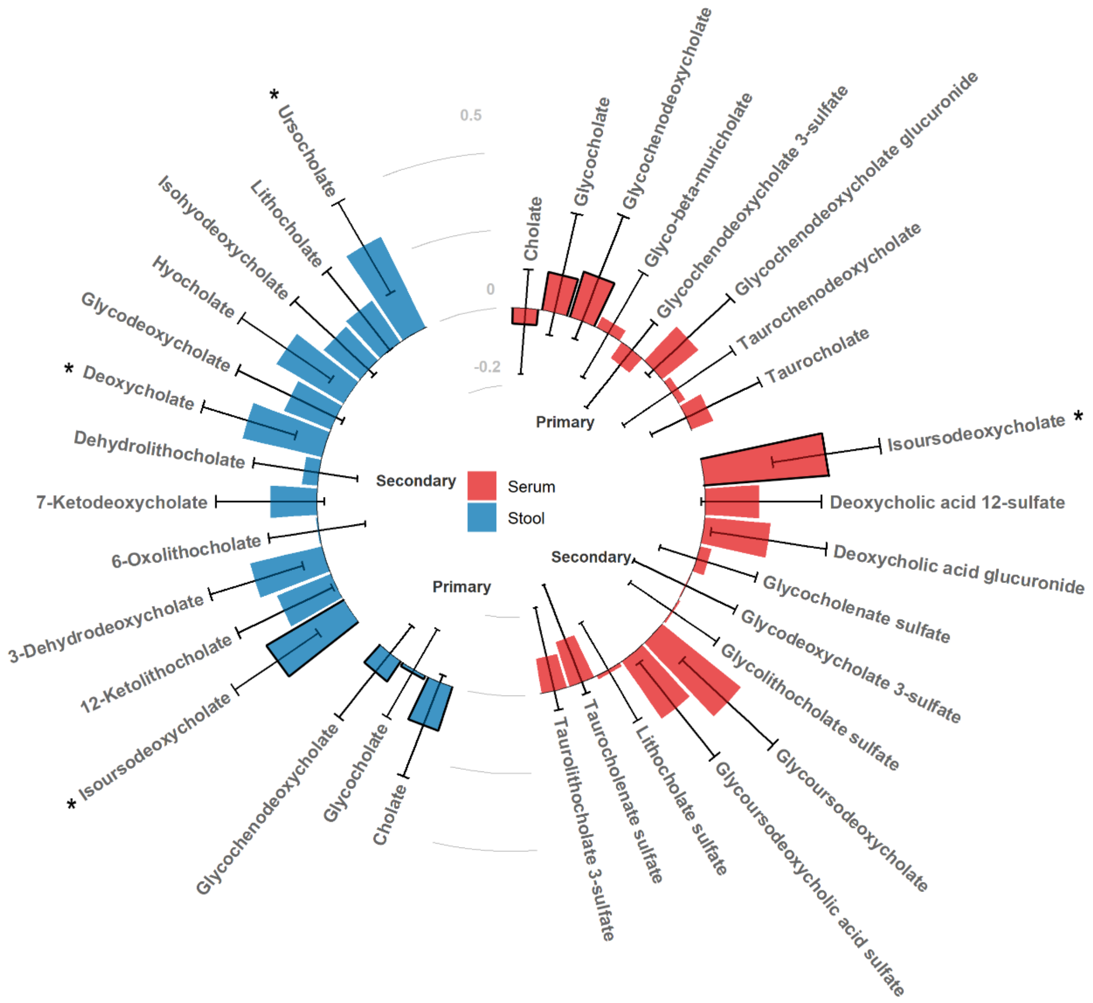

Supplementary Figure 3. Correlation of primary and secondary bile acids measured from serum and stool samples and postprandial lipaemia in PREDICT. * denotes bile acids that passed multiple testing (FDR <0.05). Bars with thick black borders represent bile acids detected in both serum and stool samples.

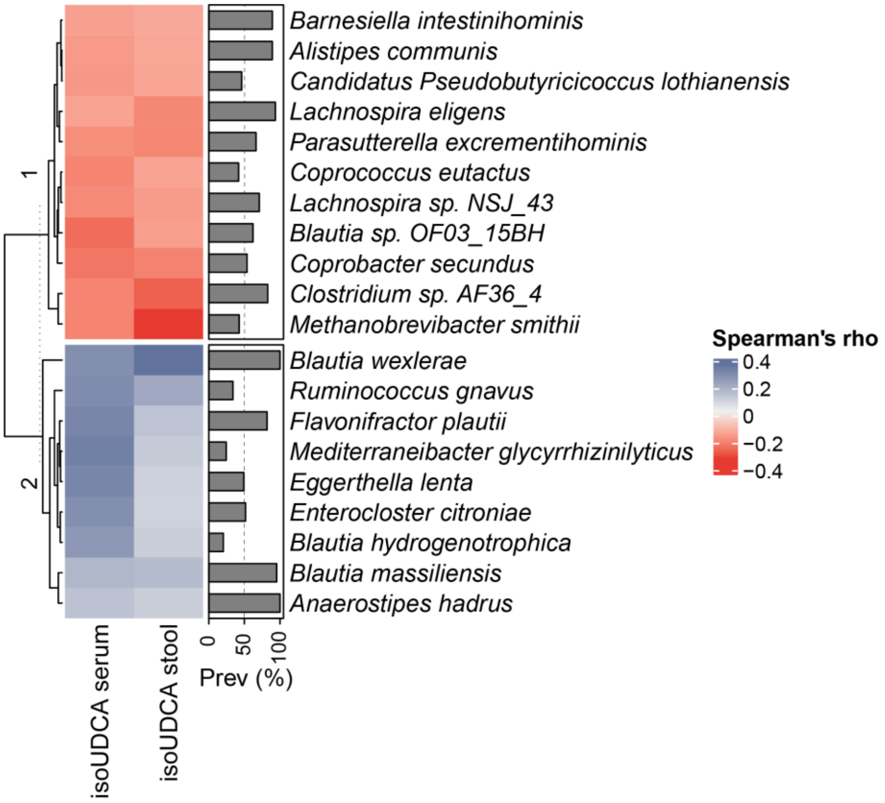

Supplementary Figure 4. Partial Spearman's correlations adjusted for age and BMI between isoUDCA levels in serum and stool, and single microbial species in 327 women from ZOE PREDICT-1. Only the characterised species with a prevalence >20% that had significant correlations (p-value<0.05), and presented the same directional effects in TwinsUK and ZOE PREDICT-1 are shown. Species are hierarchically clustered (complete linkage, Euclidean distance).

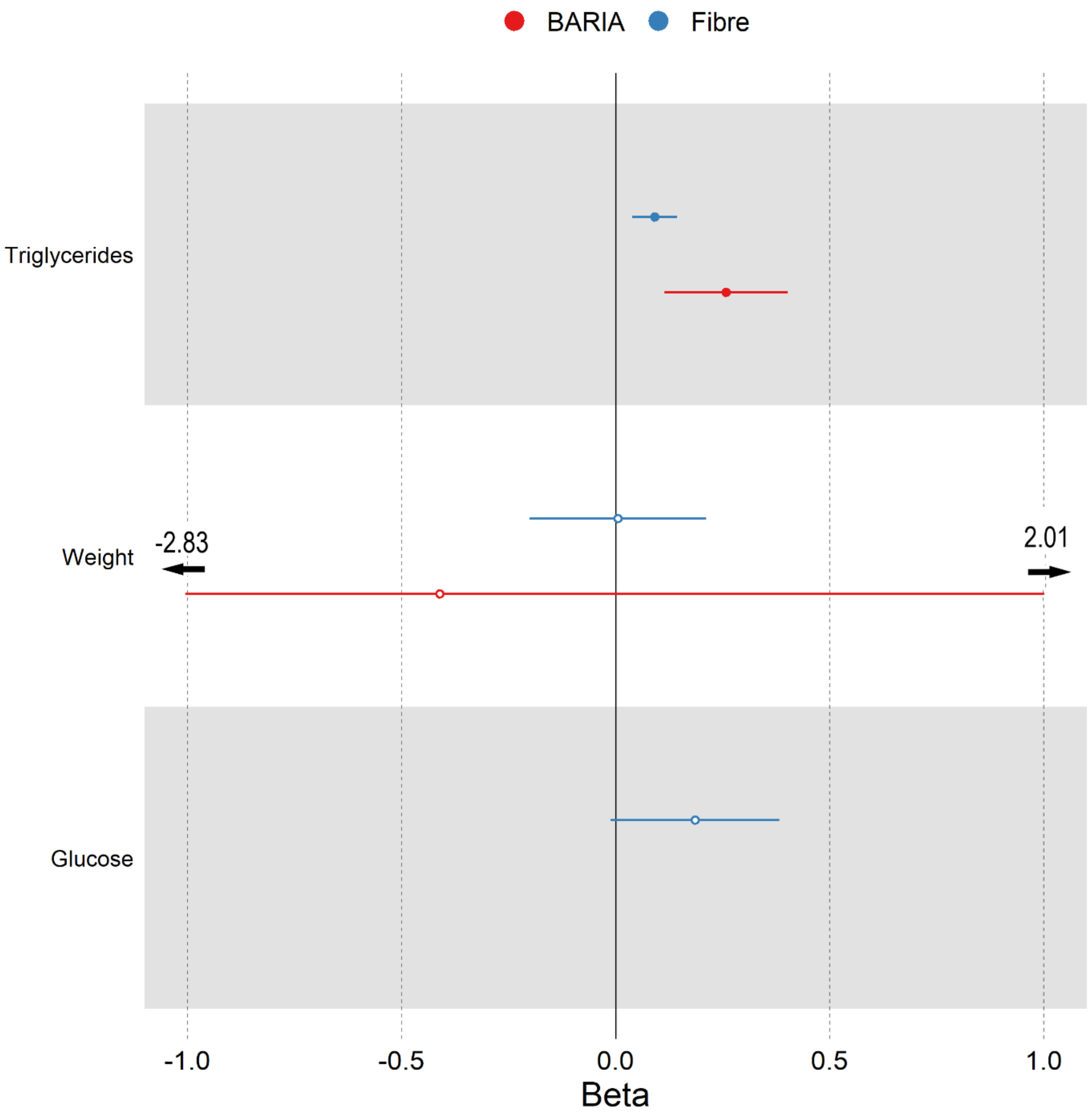

Supplementary Figure 5. Correlation between change in isoUDCA and changes in triglycerides, body weight, and glucose, in the BARIA trial and in the fibre arm of the Omega-3 and Fibre Intervention Study to Improve Metabolic Health study. Points represent regression coefficients (solid if statistically significant) adjusting for age, and BMI, and tails illustrate 95% confidence intervals.
